# The immediate effect of individual manipulation techniques on pulmonary function measures in persons with chronic obstructive pulmonary disease

**DOI:** 10.1186/1750-4732-3-9

**Published:** 2009-10-08

**Authors:** Donald R Noll, Jane C Johnson, Robert W Baer, Eric J Snider

**Affiliations:** 1 Academic Medicine Inc, 800 W Jefferson St, Kirksville, MO 63501, USA; 2 A.T. Still Research Institute, A.T. Still University, 800 W Jefferson St, Kirksville, MO 63501, USA; 3 Department of Physiology, A.T. Still University, 800 W Jefferson St, Kirksville, MO 63501, USA; 4 Department of Osteopathic Manipulative Medicine, A.T. Still University, 800 W Jefferson St, Kirksville, MO 63501, USA

## Abstract

**Background:**

The use of manipulation has long been advocated in the treatment of chronic obstructive pulmonary disease (COPD), but few randomized controlled clinical trials have measured the effect of manipulation on pulmonary function. In addition, the effects of individual manipulative techniques on the pulmonary system are poorly understood. Therefore, the purpose of this study was to determine the immediate effects of four osteopathic techniques on pulmonary function measures in persons with COPD relative to a minimal-touch control protocol.

**Methods:**

Persons with COPD aged 50 and over were recruited for the study. Subjects received five, single-technique treatment sessions: minimal-touch control, thoracic lymphatic pump (TLP) with activation, TLP without activation, rib raising, and myofascial release. There was a 4-week washout period between sessions. Protocols were given in random order until all five techniques had been administered. Pulmonary function measures were obtained at baseline and 30-minutes posttreatment. For the actual pulmonary function measures and percent predicted values, Wilcoxon signed rank tests were used to test within-technique changes from baseline. For the percent change from baseline, Friedman tests were used to test for between-technique differences.

**Results:**

Twenty-five subjects were enrolled in the study. All four tested osteopathic techniques were associated with adverse posttreatment changes in pulmonary function measures; however, different techniques changed different measures. TLP with activation increased posttreatment residual volume compared to baseline, while TLP without activation did not. Side effects were mild, mostly posttreatment chest wall soreness. Surprisingly, the majority of subjects believed they could breathe better after receiving osteopathic manipulation.

**Conclusion:**

In persons with COPD, TLP with activation, TLP without activation, rib raising, and myofascial release mildly worsened pulmonary function measures immediately posttreatment relative to baseline measurements. The activation component of the TLP technique appears to increase posttreatment residual volume. Despite adverse changes in pulmonary function measures, persons with COPD subjectively reported they benefited from osteopathic manipulation.

## Background

Chronic obstructive pulmonary disease (COPD) is a major cause of morbidity and mortality worldwide and is becoming more prevalent [1]. The Global Initiative for Chronic Obstructive Lung Disease (GOLD) defines COPD "by airflow limitation that is not fully reversible. The airflow limitation is usually progressive and associated with an abnormal inflammatory response of the lung to noxious particles or gasses [1]." The GOLD statement adds that "chronic airflow limitation characteristic of COPD is caused by a mixture of small airway disease (obstructive bronchiolitis) and parenchymal destruction (emphysema), the relative contributions of which vary from person to person [1]."

Using manipulation to treat COPD has a long tradition in the osteopathic and chiropractic professions but remains a novel approach in the larger biomedical community [2-4]. Only a few studies have attempted to measure the effects of manipulation using pulmonary function testing. Ortley et al [5] found that osteopathic manipulation caused no immediate change in pulmonary function measures in normal healthy subjects. A crossover, controlled clinical trial of asthma patients treated with chiropractic spinal manipulation twice weekly over 4 weeks found no change in either forced expiratory volume in 1 second (FEV_1_) or forced vital capacity (FVC) [6]. In postoperative cholecystectomy patients, the thoracic lymphatic pump technique was associated with a more rapid return to preoperative baseline in FEV_1 _and FVC on postoperative days 2 and 3, compared to an incentive spirometry control group [7]. However, patients with mild COPD who received twice weekly mobilization of musculoskeletal restrictions plus the thoracic lymphatic pump for an unspecified number of weeks showed no change in pulmonary function measures [8]. A well designed study of 17 subjects with more advanced COPD treated over 9 to 12 months found osteopathic manipulative treatment (OMT) significantly improved a non-validated severity of illness score, which was calculated using arterial blood gases and pulmonary function measures [9]. The use of exercises to stretch respiratory muscles in persons with COPD has been shown to improve chest wall mobility, vital capacity, and dyspnea [10].

To determine the immediate effect of a multitechnique osteopathic manipulative protocol in elderly persons with COPD, Noll et al [11] conducted a controlled clinical trial of 35 subjects who were randomized to receive either a single, 20-minute light-touch protocol or a multitechnique OMT protocol. The multitechnique protocol comprised seven techniques: soft tissue to the paraspinal thoracic muscles, rib raising, myofascial release to the abdominal diaphragm, occipital decompression, myofascial release to the thoracic inlet, pectoral traction, and the thoracic lymphatic pump with activation. Somatic dysfunction not addressed by the standardized protocol was treated with the operator's choice of myofascial release, muscle energy, or high-velocity low-amplitude techniques [11]. This study found a statistically significant 30-minute posttreatment decrease in forced expiratory flow volume at 25% (FEF_25%_) and forced expiratory flow volume at 25-75% (FEF_25-75%_) in the OMT group. The OMT group also showed a posttreatment increase in inspiratory capacity (IC), residual volume (RV), total lung capacity (TLC), and the RV/TLC ratio. These findings suggest an overall worsening of air trapping in elderly persons with COPD immediately following a multitechnique osteopathic manipulative protocol [11].

The effects of individual manipulative techniques on the pulmonary system remain poorly understood. The principal limitation of a multitechnique OMT protocol is that the contribution of each individual technique to the final result is unknown. While one technique may have a beneficial effect, another may have a detrimental effect. There remains a need to clarify the effects of individual techniques. It is not certain that use of a single isolated manipulative technique can change posttreatment pulmonary function measures. Understanding the effects of individual techniques could lead to better protocols and modifications of individual techniques to improve efficacy. For example, the multitechnique study discussed above [11] used the thoracic lymphatic pump technique with activation, denoting the sudden removal of the hands from the chest wall to trigger negative intrathoracic pressure resulting in a sudden rush of air into the lungs. It is possible that activation may worsen residual volumes in persons with COPD by enhancing air trapping and, thus, is relatively contraindicated in this population.

Therefore, we conducted a randomized controlled trial to investigate the immediate effects of individual osteopathic manipulative techniques on pulmonary function measures in persons with COPD. Four techniques were evaluated: thoracic lymphatic pump (TLP) with activation, TLP without activation, rib raising, and myofascial release. A minimal-touch treatment session was included in the study as a control. Our primary hypothesis was that the osteopathic techniques would cause measurable changes in pulmonary function measures in persons with COPD immediately following one treatment session. A secondary hypothesis was that TLP with activation would increase posttreatment residual volumes, while TLP without activation would not. Subjects were asked about side effects and their perceptions of the administered technique the day after each treatment session.

## Methods

Persons aged 50 years and over with a history of COPD were recruited for the study. The inclusion criterion was an FEV_1_/FVC ratio of 70% or less of the predicted value. Potential subjects were identified by chart review or screening office spirometry. Exclusion criteria were osteopathic or chiropractic manipulation in the previous three months, acute illness, active respiratory tract infection, acute bone fracture, inability to cooperate, thoracic scoliosis greater than 25 degrees, or chest wall deformity. Study treatments were given in an outpatient office setting in the department of Internal Medicine at A.T. Still University's Kirksville College of Osteopathic Medicine in Kirksville, Missouri between June 2003 and January 2004. Individuals were recruited from the department's clinical practice, newspaper advertisement, and speaking engagements on local talk radio and with COPD support groups. Pulmonary function testing was conducted in the pulmonary function laboratory at Northeast Regional Medical Center. Subjects were transported by wheelchair through connecting indoor hallways between the office setting and the pulmonary function laboratory to avoid fatigue. The study was approved by the local institutional review board, and all subjects gave informed consent.

Subjects received 5 single session treatment protocols in random order until all 5 protocols had been given. The duration of each treatment protocol was 5 minutes, except the myofascial technique protocol which was 5 to 10 minutes in duration. Between each treatment session, there was a 4-week washout period. The standardized treatment protocols were administered as described below:

1) Minimal-touch control: The subject took five deep breaths while the physician auscultated the lungs; then the physician purposely auscultated the heart. No attempt was made to represent the minimal-touch protocol as an osteopathic manipulative technique. Throughout the treatment session, the physician engaged the subject in an empathetic discussion of issues related to health and COPD.

2) Thoracic lymphatic pump with activation: The physician's hands were placed on the thoracic wall with the thenar eminence of each hand over the pectoralis muscles just below the clavicles; the fingers were spread and angled toward the sides of the subject's body to evenly distribute contact pressure across the chest wall. The subject took a deep breath in and exhaled. During exhalation, rhythmic, pumping action was induced by alternating pressure on the chest wall. At the end of exhalation, some residual contact pressure was maintained on the chest wall, and the subject was told to take another deep breath. This procedure was repeated several times, each time building a little more pressure on the thoracic wall. On the fourth or fifth inhalation and during the first one-third of the inhalation, the hands were *very briskly *removed from the chest wall. This removal causes a sudden release of the pressure built up in the chest wall. The rib cage springs open, and a sudden increase in thoracic negative pressure produces a rush or sucking of air into the lungs.

3) Thoracic lymphatic pump without activation: The physician's hands were placed on the thoracic wall with the thenar eminence of each hand over the pectoralis muscles just below the clavicles; the fingers were spread and angled toward the sides of the subject's body to evenly distribute pressure across the chest wall. The subject took a deep breath in and exhaled. During exhalation, rhythmic, pumping action was induced by alternating pressure on the chest wall. At the end of exhalation, some pressure was maintained on the chest wall, and the subject was told to take another deep breath. Thus, inhalation was taken against some pressure on the chest wall. At the end of three or four breathing cycles, the hands were *slowly *withdrawn from the chest wall to avoid a sudden change in chest wall pressures.

4) Rib raising: This technique was done with the subject in the supine position. The physician stood or sat at the subject's side. The physician's hands were placed under the subject's thorax, contacting the rib angles with the pads of the fingers. The fingers were flexed, and traction was applied to the rib angle. While traction was maintained, the physician used their arm as a fulcrum and kept their wrists straight; rib angles were raised anteriorly. After this cycle was repeated a number of times and the ribs in that section had improved mobility, the hands were moved up the thoracic cage, and another section was treated. This procedure was repeated until all the ribs on one side of the subject were treated. The procedure was then repeated on the subject's alternate side.

5) Myofascial release: The physician treated any myofascial structural asymmetry or restriction found in the abdominal diaphragm, the thoracic rib cage, the thoracic inlet, or the cervical region with myofascial release. Myofascial release was performed by the physician placing their hands on the subject's body, palpating for the direction in which the tissues moved most easily, moving the tissues in that direction, and holding and feeling for a release or relaxation of the tissues. The duration of the myofascial protocol session was 5 to 10 minutes because the technique takes longer to administer compared to the other techniques.

Pulmonary function measures were obtained at baseline and 30-minutes posttreatment. Pulmonary function measures included forced vital capacity (FVC) in liters, forced expiratory volume in 1 second (FEV_1_) in liters, the FEV_1_/FVC ratio in percent, the average forced expiratory flow rate over the middle 50% of the FVC (FEF _25-75%_) in L/s, the maximum forced expiratory flow rate (FEF_max_) in L/s, expiratory time in seconds, and maximal voluntary volume (MVV) in L/min. Lung volume measures were slow vital capacity (SVC) in liters, inspiratory capacity (IC) in liters, expiratory reserve volume (ERV) in liters, total gas volume (TGV) in liters, residual volume (RV) in liters, total lung capacity (TLC) in liters, and the RV/TLC ratio as a percent. Airway resistance (Raw) in cm H_2_O/L/s was also measured. Subjects were surveyed regarding possible side effects and of treatment perceptions by telephone the day after each treatment session.

To ensure the quality of measurements, certified respiratory therapists conducted the pulmonary function tests and used the American Thoracic Society criteria for test reproducibility. Pulmonary function measures were obtained with a MedGraphics^® ^1085 Series™, which measures lung volumes using plethysmography. The two osteopathic physicians who administered the treatments reviewed and practiced the study techniques together prior to enrolling subjects.

The osteopathic physicians and subjects were not blinded to the order of the techniques assessed during the treatment sessions. Subjects were told that their participation as a volunteer would include five treatment sessions, each with before and after measures of pulmonary function, once every four weeks for approximately five months. They were told the purpose of the project was to determine the effectiveness of four manipulative treatment techniques for improving breathing and pulmonary function in persons with chronic obstructive pulmonary disease. Potential participants were told one of the sessions would be a minimal-touch treatment. It was explained that even minimal-touch may have beneficial effects, which is why it was included in the study. While no special effort was made to blind subjects to the treatment they received (since this was not practical), no treatment session was represented as being more beneficial than another. Individuals involved with collecting the data, conducting the pulmonary function testing, and performing the telephone survey were blinded to the specific technique performed during each testing session.

Sample size was determined using data from a previous study [11]. The standard deviation of the total lung capacity percent predicted value was estimated to be 25%. Using a repeated measures analysis of variance (ANOVA), a sample size of 25 has power of 0.80 (α = 0.05) to detect a difference between two of the protocols of at least 9% or 0.36 standard deviations. The study statistician used blocked randomization to assign subjects to one of five treatment sequences, with randomization balanced after five subjects. Additionally, treatments were randomly assigned to one of two treatment providers. The treatment sequence assignment slips were placed in sealed envelopes and were opened by one of the treatment providers after enrollment of a new subject.

Wilcoxon signed rank tests were used to test for baseline to posttreatment changes on the actual pulmonary function measures and percent predicted values for each of the five techniques. To compare the five techniques on their immediate effects on pulmonary function measures, Friedman tests were used on the percent change from baseline to posttreatment with corresponding multiple comparisons when appropriate. Subjects were included in all comparisons for which they had complete data. *P *values less than .05 were considered statistically significant. Cohen's d was used to estimate effect sizes.

## Results

### Participants

**Figure 1:**
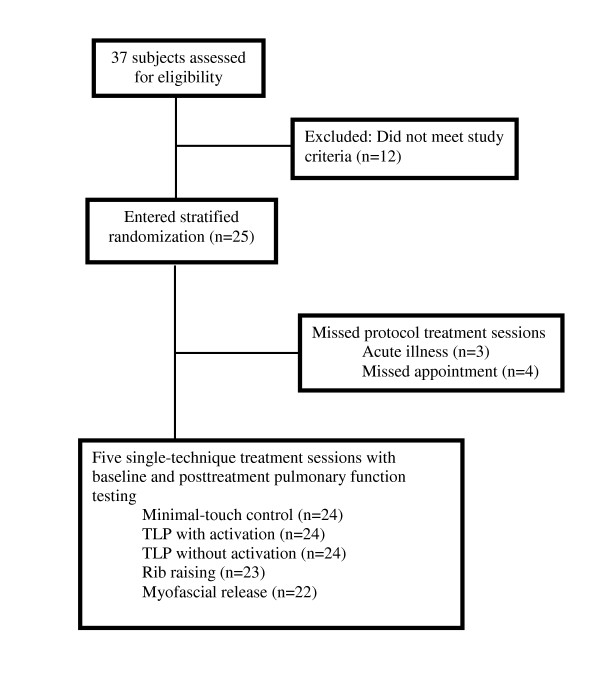
**Study outline**.

Twenty-five subjects were enrolled in the study (Figure 1). Two subjects missed treatment sessions because of acute illness; one missed one session due to a hospitalized spouse and one missed two sessions due to an acute episode of pneumonia requiring hospitalization. One subject missed four of the five treatment sessions because of poor compliance. None of the subjects experienced a COPD exacerbation episode following any of the treatments sessions. Subjects were aged 51 to 80 years with a mean ± SD age of 68 ± 8 years. All subjects were Caucasian. Demographic characteristics, including sex, smoking habits, prior experience with manipulation, current medical therapies for COPD, and common comorbid medical conditions, are presented in Table 1.

**Table 1:** Demographic characteristics of subjects (N = 25)*

**Variable**	**Results**	**Variable**	**Results**
Sex		Atrovent updraft	
Female	11 (44%)	Yes	8 (35%)
Male	14 (56%)	No	15 (65%)

Presently smoking		Type II DM	
Yes	9 (36%)	Yes	4 (16%)
No	16 (64%)	No	21 (84%)

Prior OMT		History of stroke	
Yes	21 (84%)	Yes	2 (8%)
No	4 (16%)	No	23 (92%)

Prior chiropractic treatment		History of heart failure	
Yes	22 (88%)	Yes	2 (8%)
No	3 (12%)	No	23 (92%)

Preference: OMT or chiropractic		History of thyroid disease	
OMT	11 (58%)	Yes	3 (12%)
No preference	8 (42%)	No	22 (88%)

Manipulation treatment for respiratory problem		History of pulmonary tuberculosis	
Yes	14 (56%)	No	25 (100%)
No	11 (44%)		

Frequency of manipulative treatment		History of hypertension	
More than 1 per year	4 (16%)	Yes	7 (28%)
One or less per year	21 (84%)	No	18 (72%)

Oxygen use		History of coronary heart disease	
Yes	12 (50%)	Yes	7 (28%)
No	12 (50%)	No	18 (72%)

Theophylline use		History of pneumonia	
Yes	2 (8%)	Yes	20 (80%)
No	22 (92%)	No	5 (20%)

Inhaled steroid use		History of asthma	
Yes	10 (42%)	Yes	11 (44%)
No	14 (58%)	No	14 (56%)

Oral steroid use		History of cancer	
Yes	6 (26%)	Yes	10 (40%)
No	17 (74%)	No	15 (60%)

Inhaled bronchodilator		History of CABG	
Yes	18 (75%)	Yes	2 (8%)
No	6 (25%)	No	22 (92%)

Updraft treatments		History of lung surgery	
Yes	13 (52%)	Yes	2 (8%)
No	12 (48%)	No	23 (92%)

Atrovent MDI		Family history of lung disease	
Yes	14 (58%)	Yes	13 (54%)
No	10 (42%)	No	11 (46%)

*Data are presented as number (% of subjects) for variables. Total counts may not add up to N due to missing values. All subjects were Caucasian.

OMT, osteopathic manipulative treatment; MDI, metered-dose inhaler; DM, diabetes mellitus; CABG, coronary artery bypass graft.

### Pulmonary function measures

The actual pulmonary function results at baseline and 30-minutes posttreatment are summarized in Table 2. Measures with statistically significant changes from baseline to posttreatment are highlighted. For the minimal-touch control protocol, only IC (d = 0.57) showed a posttreatment decrease from baseline. TLP with activation had posttreatment decreases from baseline in FEF_max _(d = 0.75), MVV (d = 0.59), SVC (d = 0.45), and ERV (d = 0.97); and posttreatment increases from baseline in RV (d = 0.30) and the RV/TLC ratio (d = 0.31). For TLP without activation, posttreatment FVC (d = 0.29), FEF_25-75% _(d = 0.38), and MVV (d = 0.52) decreased relative to baseline and airway resistance (d = 0.30) increased relative to baseline. Consistent with the study hypothesis, TLP with activation increased posttreatment RV compared to baseline (d = 0.30), and TLP without activation did not (d = 0.12). Rib raising was associated with a posttreatment decrease from baseline in FEF_max _(d = 0.55) and MVV (d = 0.75). Myofascial release was associated with a posttreatment decrease from baseline in FEV_1 _(d = 0.53), FEF_25-75% _(d = 0.47), FEF_max _(d = 0.60), MVV (d = 0.53), and SVC (d = 0.56).

**Table 2:** Baseline and posttreatment actual pulmonary function results for each technique

**Technique**	**Minimal-touch Control**	**TLP with Activation**	**TLP Without Activation**	**Rib Raising**	**Myofascial Release**
FVC (L)	2.79 ± 0.99	2.83 ± 1.05	**2.91 ± 1.05**	2.75 ± 1.02	2.83 ± 1.02
	2.80 ± 0.97	2.79 ± 1.00	**2.85 ± 1.01**	2.77 ± 1.05	2.79 ± 0.97
	*P *= 0.65	*P *= 0.31	***P *= 0.02**	*P *= 0.85	*P *= 0.12
FEV_1 _(L)	1.57 ± 0.79	1.59 ± 0.82	1.63 ± 0.78	1.51 ± 0.79	**1.56 ± 0.75**
	1.57 ± 0.79	1.58 ± 0.81	1.59 ± 0.75	1.53 ± 0.82	**1.52 ± 0.71**
	*P *= 0.90	*P *= 0.40	*P *= 0.07	*P *= 0.45	***P *= 0.03**
FEV_1_/FVC (%)	55 ± 13	54 ± 13	55 ± 13	53 ± 12	54 ± 12
	54 ± 13	55 ± 14	55 ± 13	54 ± 13	53 ± 11
	*P *= 0.67	*P *= 0.73	*P *= 0.63	*P *= 0.46	*P *= 0.46
FEF_25-75% _(L/s)	0.81 ± 0.62	0.80 ± 0.61	**0.82 ± 0.55**	0.73 ± 0.52	**0.75 ± 0.52**
	0.78 ± 0.62	0.81 ± 0.59	**0.75 ± 0.51**	0.77 ± 0.62	**0.68 ± 0.46**
	*P *= 0.26	*P *= 0.30	***P *= 0.006**	*P *= 0.56	***P *= 0.007**
FEF_max _(L/s)	4.55 ± 2.28	**4.75 ± 2.34**	4.79 ± 2.35	**4.68 ± 2.35**	**4.69 ± 2.31**
	4.41 ± 2.32	**4.43 ± 2.16**	4.55 ± 2.27	**4.37 ± 2.31**	**4.42 ± 2.37**
	*P *= 0.38	***P *= 0.001**	*P *= 0.08	***P *= 0.01**	***P *= 0.007**
Expiratory time (s)	9.60 ± 1.63	9.54 ± 1.62	9.79 ± 2.52	10.00 ± 1.90	9.78 ± 1.74
	9.74 ± 1.77	9.99 ± 2.60	9.29 ± 1.95	10.37 ± 2.08	10.06 ± 2.01
	*P *= 0.53	*P *= 0.65	*P *= 0.055	*P *= 0.27	*P *= 0.42
MVV (L/min)	56 ± 30	**58 ± 29**	**60 ± 31**	**56 ± 31**	**58 ± 30**
	56 ± 30	**55 ± 29**	**57 ± 30**	**52 ± 28**	**55 ± 30**
	*P *= 0.64	***P *= 0.005**	***P *= 0.02**	***P *= 0.0004**	***P *= 0.03**
SVC (L)	2.91 ± 1.05	**2.98 ± 1.08**	3.05 ± 1.10	2.82 ± 1.05	**3.07 ± 1.09**
	2.90 ± 1.14	**2.87 ± 1.04**	2.98 ± 1.10	2.85 ± 1.12	**2.95 ± 1.06**
	*P *= 0.42	***P *= 0.04**	*P *= 0.09	*P *= 0.81	***P *= 0.008**
IC (L)	**2.14 ± 0.78**	2.10 ± 0.80	2.13 ± 0.79	2.00 ± 0.73	2.14 ± 0.77
	**2.03 ± 0.72**	2.12 ± 0.76	2.09 ± 0.76	2.06 ± 0.78	2.10 ± 0.76
	***P *= 0.008**	*P *= 0.42	*P *= 0.38	*P *= 0.84	*P *= 0.19
ERV (L)	0.77 ± 0.44	**0.88 ± 0.45**	0.92 ± 0.51	0.82 ± 0.52	0.93 ± 0.55
	0.87 ± 0.62	**0.75 ± 0.44**	0.89 ± 0.64	0.79 ± 0.52	0.86 ± 0.48
	*P *= 0.18	***P*<0.0001**	*P *= 0.22	*P *= 0.42	*P *= 0.30
TGV (L)	4.14 ± 0.91	4.17 ± 0.88	4.29 ± 0.97	4.32 ± 1.13	4.35 ± 1.05
	4.07 ± 0.90	4.18 ± 0.91	4.23 ± 1.03	4.16 ± 1.05	4.33 ± 1.08
	*P *= 0.43	*P *= 0.56	*P *= 0.16	*P *= 0.19	*P *= 0.91
RV (L)	3.36 ± 0.81	**3.30 ± 0.77**	3.38 ± 0.92	3.50 ± 1.21	3.41 ± 0.96
	3.19 ± 0.84	**3.41 ± 0.93**	3.33 ± 0.99	3.37 ± 1.01	3.48 ± 1.08
	*P *= 0.15	***P *= 0.03**	*P *= 0.48	*P *= 0.62	*P *= 0.34
TLC (L)	6.27 ± 1.16	6.27 ± 1.14	6.41 ± 1.11	6.32 ± 1.31	6.47 ± 1.23
	6.10 ± 1.03	6.29 ± 0.99	6.33 ± 1.21	6.21 ± 1.14	6.44 ± 1.27
	*P *= 0.07	*P *= 0.41	*P *= 0.22	*P *= 0.39	*P *= 0.34
RV/TLC (%)	54 ± 12	**53 ± 12**	53 ± 13	55 ± 13	53 ± 13
	53 ± 13	**55 ± 14**	53 ± 13	55 ± 14	54 ± 13
	*P *= 0.59	***P *= 0.04**	*P *= 0.79	*P *= 0.85	*P *= 0.058
Raw (cm H_2_O/L/s)	4.09 ± 3.18	3.94 ± 3.15	**3.36 ± 2.19**	4.30 ± 3.09	4.44 ± 3.17
	4.05 ± 2.76	3.94 ± 2.74	**4.04 ± 3.20**	4.22 ± 3.11	4.06 ± 2.75
	*P *= 0.95	*P *= 0.82	***P *= 0.04**	*P *= 0.30	*P *= 0.75

Results are reported as mean ± SD. Wilcoxon signed rank tests were used to test for significant baseline to posttreatment within-technique changes. Measures with significant within-technique changes are highlighted.

FVC, forced vital capacity; FEV_1_, forced expiratory volume in 1 second; FEF_25-75%_, average forced expiratory flow rate over the middle 50% of the FVC; FEF_max_, maximum forced expiratory flow rate; MVV, maximal voluntary volume; SVC, slow vital capacity; IC, inspiratory capacity; ERV, expiratory reserve volume; TGV, total gas volume; RV, residual volume; TLC, total lung capacity; Raw, airway resistance.

The mean percent predicted values at baseline and 30-minutes posttreatment are summarized in Table 3. Statistically significant changes from baseline to posttreatment are highlighted. The minimal-touch control was associated with significant decreases in both IC (d = 0.55) and TLC (d = 0.42). TLP with activation decreased the percent predicted values for FEF_max _(d = 0.75), MVV (d = 0.58), SVC (d = 0.48), and ERV (d = 0.97) and increased the percent predicted value for RV (d = 0.31). TLP without activation decreased the FVC (d = 0.28), FEV_1 _(d = 0.34), FEF_25-75% _(d = 0.39), FEF_max _(d = 0.51), and MVV (d = 0.53), and increased airway resistance (d = 0.29). Similar to the actual pulmonary function results, TLP with activation increased the percent predicted RV (d = 0.31), while TLP without activation did not (d = 0.10). Rib raising decreased the percent predicted values for FEF_max _(d = 0.65) and MVV (d = 0.74). Myofascial release decreased the percent predicted values for FEV_1 _(d = 0.52), FEF_25-75% _(d = 0.49), FEF_max _(d = 0.49), MVV (d = 0.56), and SVC (d = 0.60).

**Table 3:** Baseline and posttreatment percent predicted values for each technique

**Technique**	**Minimal-touch Control**	**TLP with Activation**	**TLP Without Activation**	**Rib Raising**	**Myofascial Release**
FVC	82 ± 22	83 ± 23	**84 ± 24**	81 ± 22	82 ± 24
	82 ± 21	82 ± 21	**83 ± 23**	82 ± 22	81 ± 23
	*P *= 0.58	*P *= 0.37	***P *= 0.03**	*P *= 0.65	*P *= 0.26
FEV_1_	58 ± 25	58 ± 24	**59 ± 25**	56 ± 23	**57 ± 25**
	58 ± 24	58 ± 24	**58 ± 24**	57 ± 25	**56 ± 24**
	*P *= 0.83	*P *= 0.37	***P *= 0.04**	*P *= 0.39	***P *= 0.04**
FEF_25-75%_	29 ± 18	28 ± 17	**29 ± 18**	26 ± 16	**27 ± 17**
	28 ± 18	29 ± 18	**27 ± 17**	27 ± 19	**24 ± 16**
	*P *= 0.31	*P *= 0.30	***P *= 0.006**	*P *= 0.57	***P *= 0.009**
FEF_max_	70 ± 29	**73 ± 28**	**72 ± 30**	**73 ± 30**	**71 ± 30**
	68 ± 28	**68 ± 27**	**68 ± 29**	**68 ± 29**	**67 ± 31**
	*P *= 0.29	***P *= 0.002**	***P *= 0.04**	***P *= 0.006**	***P *= 0.009**
MVV	52 ± 23	**53 ± 21**	**55 ± 24**	**52 ± 23**	**53 ± 24**
	52 ± 24	**51 ± 21**	**52 ± 24**	**49 ± 21**	**51 ± 23**
	*P *= 0.75	***P *= 0.006**	***P *= 0.02**	***P *= 0.0006**	***P *= 0.02**
SVC	79 ± 20	**81 ± 20**	81 ± 23	77 ± 19	**81 ± 22**
	78 ± 22	**78 ± 20**	79 ± 22	78 ± 21	**78 ± 21**
	*P *= 0.41	***P *= 0.04**	*P *= 0.07	*P *= 0.65	***P *= 0.01**
IC	**79 ± 23**	76 ± 21	77 ± 24	74 ± 19	77 ± 23
	**75 ± 21**	78 ± 21	75 ± 21	76 ± 21	76 ± 21
	***P *= 0.01**	*P *= 0.34	*P *= 0.34	*P *= 0.76	*P *= 0.22
ERV	80 ± 35	**94 ± 37**	97 ± 48	87 ± 40	93 ± 45
	88 ± 47	**78 ± 34**	92 ± 55	82 ± 40	87 ± 39
	*P *= 0.22	***P *< 0.0001**	*P *= 0.22	*P *= 0.42	*P *= 0.22
TGV	130 ± 30	131 ± 31	134 ± 34	136 ± 34	134 ± 33
	127 ± 29	131 ± 31	132 ± 34	131 ± 34	134 ± 33
	*P *= 0.47	*P *= 0.62	*P *= 0.18	*P *= 0.21	*P *= 0.99
RV	150 ± 41	**147 ± 38**	151 ± 45	156 ± 54	152 ± 46
	143 ± 42	**152 ± 44**	149 ± 48	151 ± 48	155 ± 48
	*P *= 0.17	***P *= 0.04**	*P *= 0.51	*P *= 0.59	*P *= 0.35
TLC	**107 ± 16**	106 ± 15	108 ± 17	108 ± 17	108 ± 16
	**104 ± 15**	107 ± 15	106 ± 18	106 ± 16	108 ± 17
	***P *= 0.048**	*P *= 0.44	*P *= 0.16	*P *= 0.45	*P *= 0.36
Raw	246 ± 175	234 ± 170	**205 ± 124**	249 ± 192	275 ± 199
	243 ± 151	237 ± 157	**248 ± 202**	250 ± 167	247 ± 153
	*P *= 0.89	*P *= 0.81	***P *= 0.046**	*P *= 0.28	*P *= 0.83

Results are reported as mean ± SD. Wilcoxon signed rank tests were used to analyze for significant baseline to posttreatment within-technique changes. Measures with significant within-technique changes are highlighted.

FVC, forced vital capacity; FEV_1_, forced expiratory volume in 1 second; FEF_25-75%_, average forced expiratory flow rate over the middle 50% of the FVC; FEF_max_, maximum forced expiratory flow rate; MVV, maximal voluntary volume; SVC, slow vital capacity; IC, inspiratory capacity; ERV, expiratory reserve volume; TGV, total gas volume; RV, residual volume; TLC, total lung capacity; Raw, airway resistance.

The mean percent change values for each of the five techniques are presented in Table 4. There were no significant differences between the techniques on the percent change from baseline to 30-minutes posttreatment.

**Table 4:** Baseline to posttreatment percent changes

**Technique**	**Minimal-touch Control**	**TLP with Activation**	**TLP Without Activation**	**Rib Raising**	**Myofascial Release**	***P* Value**
FVC	0.7 ± 5.0	0.3 ± 11.9	-1.4 ± 8.1	0.7 ± 8.3	-0.7 ± 6.2	0.67
FEV_1_	0.6 ± 6.6	0.2 ± 7.8	-1.0 ± 7.8	1.2 ± 8.0	-1.4 ± 6.8	0.10
FEV_1_/FVC	-0.2 ± 5.3	0.3 ± 9.8	0.4 ± 4.0	0.7 ± 6.4	-0.4 ± 5.3	0.71
FEF_25-75%_	-2.4 ± 13.8	3.3 ± 23.4	-5.5 ± 21.9	0.7 ± 22.5	-7.3 ± 17.8	0.12
FEF_max_	-2.4 ± 14.5	-6.3 ± 9.3	-5.6 ± 12.6	-6.1 ± 13.7	-5.5 ± 17.3	0.25
Expiratory time	1.9 ± 11.4	5.0 ± 20.9	-3.6 ± 12.4	5.2 ± 19.0	3.5 ± 15.5	0.48
MVV	-0.0 ± 10.5	-4.9 ± 10.7	-4.6 ± 8.6	-5.7 ± 10.1	-5.7 ± 9.6	0.40
SVC	-0.3 ± 7.7	-3.2 ± 7.2	-2.0 ± 7.4	0.6 ± 15.5	-3.5 ± 6.3	0.51
IC	-3.7 ± 9.9	2.5 ± 9.2	-0.6 ± 10.6	2.7 ± 14.3	-1.2 ± 10.1	0.21
ERV	10.9 ± 38.3	-12.4 ± 31.3	-6.9 ± 19.4	3.7 ± 59.8	-1.1 ± 30.9	0.09
TGV	-1.3 ± 8.2	0.6 ± 8.6	-1.6 ± 8.0	-3.2 ± 9.1	-0.2 ± 7.2	0.19
RV	-4.1 ± 17.4	3.2 ± 14.2	-1.3 ± 12.4	-1.9 ± 13.9	2.3 ± 11.1	0.10
TLC	-2.2 ± 6.1	1.1 ± 7.2	-1.4 ± 5.9	-1.1 ± 5.7	-0.5 ± 5.3	0.36
RV/TLC	-2.4 ± 14.5	1.6 ± 10.1	-0.2 ± 7.6	-1.2 ± 10.4	2.8 ± 7.4	0.21
Raw	3.0 ± 27.5	5.9 ± 33.3	22.9 ± 46.1	1.2 ± 28.8	0.7 ± 30.9	0.53

Results are reported as mean ± SD. Friedman tests were used to test for differences between the techniques.

FVC, forced vital capacity; FEV_1_, forced expiratory volume in 1 second; FEF_25-75%_, average forced expiratory flow rate over the middle 50% of the FVC; FEF_max_, maximum forced expiratory flow rate; MVV, maximal voluntary volume; SVC, slow vital capacity; IC, inspiratory capacity; ERV, expiratory reserve volume; TGV, total gas volume; RV, residual volume; TLC, total lung capacity; Raw, airway resistance.

### Telephone survey results

Side effects reported during the posttreatment telephone survey are summarized in Table 5. There were 14 separate reports of side effects possibly associated with the study treatments. None were characterized as severe. Musculoskeletal soreness or pain was a common side effect. The longest duration of these symptoms was reported to be "two or three days." The minimal-touch control protocol had the fewest reported side effects, with only one subject reporting muscle soreness, which lasted "two or three days" following the session. The subject attributed the muscle soreness to the pulmonary function testing. TLP with activation had the highest incidence of reported side effects (19%), with TLP without activation a close second (17%). For the rib raising technique, two of the three reports indicated pain during the treatment. The myofascial release technique had two reports of side effects, one being posttreatment chest soreness and the other being rib pain during treatment near the location of an old surgery. Reported side effect rates were 6% for the minimal-touch control technique and between 13% and 19% for the osteopathic techniques.

**Table 5:** Reported study side effects surveyed by telephone the day after each treatment session*

**Technique**	**Minimal- touch Control**	**TLP with Activation**	**TLP Without Activation**	**Rib Raising**	**Myofascial Release**
Subject	A	B	C	B	D

Reported Side Effect	PFT made sore for 2 or 3 days	Chest pain in middle for 24 hours	Soreness in front chest	Chest pain during the procedure	Soreness in chest for 1 day

Subject		E	F	F	G

Reported Side Effect		Soreness in the chest, remains next day	Stiff neck and headache the next day	Tired back	During the treatment, not afterwards, sore in lower ribs near (old) surgery site

Subject		H	I	J	

Reported Side Effect		Chest pressure during treatment, pain for 2-3 days	Sore in chest later that night lasted until next day, and chest congestion	Pain during treatment, better afterwards, best so far	

Subject		K	L		

Reported Side Effect		Cramps in left lung	Discomfort across the back afterwards		

*Reported Side Effect Rate*	1 out of 18 (6%)	4 out of 23 (17%)	4 out of 21 (19%)	3 out of 20 (15%)	2 out of 16 (13%)

TLP, thoracic lymphatic pump; PFT, pulmonary function testing.

* Paraphrased from subjects' reports.

Telephone survey responses relating to the subjects' perception of the treatment sessions are summarized in Table 6. Survey responses from all study participants were not obtained, primarily due to failure to contact some subjects the day after a treatment session. In addition, a few subjects declined to answer to some questions. The minimal-touch control technique had the lowest percentage of positive responses. The majority of those subjects receiving one of the four osteopathic techniques believed their health benefited (53-76%) and they could breathe better after treatment (50-79%). When asked if they would recommend the treatment to others, at least 90% of subjects receiving one of the four osteopathic techniques said "Yes." For the minimal-touch control technique, 71% of subjects indicated they enjoyed receiving the treatment and would recommend it to others.

**Table 6:** Subject perceptions of the treatment sessions surveyed by telephone the day after each session

**Question**	**Minimal-touch Control**	**TLP with Activation**	**TLP Without Activation**	**Rib Raising**	**Myofascial Release**
Do you believe your health has benefited from the most recent manipulation treatment you received in this study?	41% (n = 17)	76% (n = 21)	67% (n = 21)	68% (n = 19)	53% (n = 15)
					
Do you feel that you breathed better after the most recent manipulation treatment you received in this study?	44% (n = 18)	74% (n = 23)	57% (n = 21)	79% (n = 19)	50% (n = 16)
					
Did you enjoy receiving the most recent manipulation treatment in this study?	71% (n = 17)	86% (n = 22)	80% (n = 20)	88% (n = 17)	88% (n = 16)
					
Would you recommend the most recent treatment to others?	71% (n = 17)	91% (n = 22)	90% (n = 20)	95% (n = 19)	94% (n = 16)

Subjects were asked to respond "Yes" or "No" to the questions in the table. Results are presented as the percentage of "Yes" responses and the total number of responses for each question.

TLP, thoracic lymphatic pump.

## Discussion

In the current study, all four osteopathic techniques mildly worsened pulmonary function measures in persons with COPD immediately following treatment. However, each technique had different effects on pulmonary function. The TLP with activation technique was associated with a posttreatment increase in RV, while the same technique without the activation component, that is TLP without activation, was not associated with a posttreatment rise in RV. This suggests that it is the activation component of the technique that causes a posttreatment increase in RV. Despite this adverse change, 74% of subjects who received TLP with activation reported the treatment helped them breathe better. Only 57% of subjects who received TLP without activation believed the treatment helped them breathe better even though RV was not adversely affected by this technique. As expected, the minimal-touch control was associated with the fewest pulmonary function changes with only the posttreatment IC and TLC being decreased. Within-technique analysis showed that all four osteopathic techniques reduced MVV for both actual and percent predicted values. The TLP with activation technique caused the largest number of adverse pulmonary function changes. Rib raising had the fewest adverse changes.

To our knowledge, this is the first study to test the immediate effects of individual osteopathic techniques on pulmonary function measures in persons with COPD. However, one of the techniques used in the current study, the TLP, has been studied more extensively than the others. The TLP technique was first described by Miller in 1927 [12]. It was specifically developed to treat pneumonia and was intended to enhance bacterial antigen absorption into the immune system through increased lymphatic circulation and, thus, stimulate a quicker antibody response [12]. Recent animal model studies have confirmed that lymphatic pumping does increase lymphatic flow through the thoracic duct [13]. Animal model studies have also shown that the concentration of leukocytes in thoracic duct lymph are significantly increased by lymphatic pumping [14]. In 1935, Morey was the first to describe activation with the TLP technique: he developed the activation component of the technique to enhance lymphatic flow [15]. The TLP with activation technique has been utilized in the protocols of several studies that examine the use of OMT in the elderly hospitalized with pneumonia [16-18]. This technique is thought to be helpful in countering pulmonary atelectasis. A study evaluating the use of TLP for atelectasis in postoperative cholecystectomy patients found a more rapid postoperative improvement in FEV_1 _and FVC in a TLP-treated group relative to a conventional incentive spirometry group [7]. However, since the technique description indicates that the resistive force on the chest wall was "gently released" toward the end of the fifth inspiration [7], the TLP technique used in this study probably did not incorporate activation. Future studies are needed to determine if the activation component of the TLP technique increases lymphatic circulation and to determine the efficacy of this technique in treating or preventing atelectasis.

Reported side effects were relatively mild and transitory. The majority were related to musculoskeletal soreness or pain either during a treatment session or following a treatment session. Vick et al [19] reviewed the safety of manipulative treatment from 1925 to 1993 and found 185 reports of serious adverse events but no published reports of minor or transitory complications. Mild to moderate side effects associated with manipulation are likely underreported and poorly characterized. In a small study of OMT in nursing home residents [20], 1 out of 7 participants (14%) who answered a posttreatment questionnaire reported musculoskeletal soreness. In a previous study that tested the immediate effects of a multitechnique osteopathic protocol in persons with COPD [11], 2 out of 15 surveyed participants (12%) reported posttreatment muscle soreness. These published side effect rates are consistent with the 13-17% reported side effect rates for each of the four osteopathic techniques of the current study.

Despite the posttreatment worsening of pulmonary function measures, the majority of subjects reported that their health benefited from treatment, that they could breathe better after each treatment, that treatments were enjoyable, and that they would recommend the treatment to others. Favorable survey responses for the minimal-touch control session were fewer, but subjects seemed to value the auscultation and the empathetic discussion of their health issues. A previous osteopathic multitechnique study also found that the majority of participants believed that they benefited from manipulative treatment, despite objective worsening of pulmonary function measures [11]. In that study [11], 71% of subjects reported their health benefited from treatment, 82% reported they could breath better after treatment, 94% said they enjoyed the treatment, and 88% reported they would recommend the treatment to others. These published results are similar to the results of the current study.

There are several mechanistic explanations for the study findings. Persons with COPD have chronic airway inflammation and are prone to bronchospasm and air trapping. The physical movement associated with the osteopathic techniques may trigger bronchospasm or loosen airway secretions, which could worsen air trapping. This problem may be limited to those with diseased airways since normal airways are likely to respond differently. The subjective sense of improvement following treatment despite worsening pulmonary function measures seems paradoxical. Belief in the treatment and the placebo effect are possible explanations. There is evidence that manipulation promotes a sense of well-being and decreases pain by modulating endocannabinoids, endogenous opioids, and serotonin [21-24]. These physiologic changes could provide a mechanistic explanation for the positive responses to the posttreatment survey.

The current study has several limitations. Pulmonary function testing was only done at baseline and approximately 30-minutes posttreatment. The duration of any treatment effects on pulmonary function measures beyond 30 minutes is unknown. It is possible that the techniques had a beneficial effect on pulmonary function measures after the initial posttreatment decline. More likely, however, the pulmonary function measures returned to baseline. We did not use functional measures, such as a 6-minute walk or serologic markers, in the current study. While the sample size was large enough to detect moderate differences between the techniques, the results should be interpreted with caution because of the relatively small sample size and because these results have not been confirmed by other studies. Blinding was limited to those collecting the data. Because no attempt was made to blind the subjects, they were not asked which session they thought was the minimal-touch control. Conclusions based on the posttreatment survey results are also limited because the survey was not a validated instrument and the responses were subjective.

The findings of this study have good external validity to persons with clinically stable COPD since the study cohort included persons with common comorbid medical conditions. However, our results have less generalizability with other conditions, such as asthma or normal lung function. Therefore, results of the current study are applicable to the techniques tested and cannot be generalized to other osteopathic techniques.

Despite the adverse changes in pulmonary function measures, the four osteopathic techniques appear to be reasonably safe for persons with stable COPD. The four techniques were shown to be "safe" in the sense that none of the study participants reported an acute exacerbation of symptoms which required a change in medical treatment or resulted in hospitalization. Also, the majority of subjects reported feeling and breathing better after treatment. Because of the small sample size, conclusions regarding safety should be made with caution. Symptomatic exacerbations are common in this population. If the number of participants had been larger, it is likely such cases would have occurred regardless of group assignment. However, in the context of an acute exacerbation of COPD, where even small transient adverse changes may have serious consequences, these techniques should be considered contraindicated. Further, the TLP with activation and myofascial release techniques may be relatively contraindicated even in persons with stable COPD since these techniques may increase RV. It is important to collect information on side effects and adverse events even in small clinical trials such as this one, so that a body of literature can be developed on the tolerability of various manipulative techniques.

## Conclusion

The four osteopathic techniques of the current study -- TLP with activation, TLP without activation, rib raising, and myofascial release -- were associated with a modest posttreatment worsening of pulmonary function measures in persons with COPD. The different techniques had different effects on the pulmonary function measures. The activation component of the TLP technique increased RV in persons with COPD. Side effects were relatively mild and transitory, and were related to posttreatment chest wall soreness. Overall, the techniques tested worsened pulmonary function at thirty minutes posttreatment in persons with COPD. Paradoxically, the majority of subjects believed they could breathe better after receiving the osteopathic treatments. The longer term effects of these techniques on pulmonary function are still not known.

## Competing interests

The authors declare that they have no competing interests.

## Authors' contributions

DRN conceived the study, participated in its design, acquired and interpreted the data, and drafted the manuscript. JCJ participated in the study design, performed the statistical analyses, and revised the manuscript for content. RWB participated in the study design, acquired and interpreted the data, and revised the manuscript for content. EJS acquired the data and revised the manuscript for content. All authors read and approved the final manuscript.
